# Inflammatory Bowel Disease Presenting With Retiform Purpura

**DOI:** 10.1097/PG9.0000000000000232

**Published:** 2022-08-16

**Authors:** Laura A. Quinn, Grant R. Plost, Bradford Siegele, Amy G. Feldman, Edwin de Zoeten

**Affiliations:** From the * Section of Pediatric Gastroenterology, Hepatology and Nutrition, University of Colorado School of Medicine, Aurora, CO; † Department of Pediatric Dermatology, University of Colorado School of Medicine, Aurora, CO; ‡ Department of Pathology, University of Colorado School of Medicine, Aurora, CO.

**FIGURE 1.**
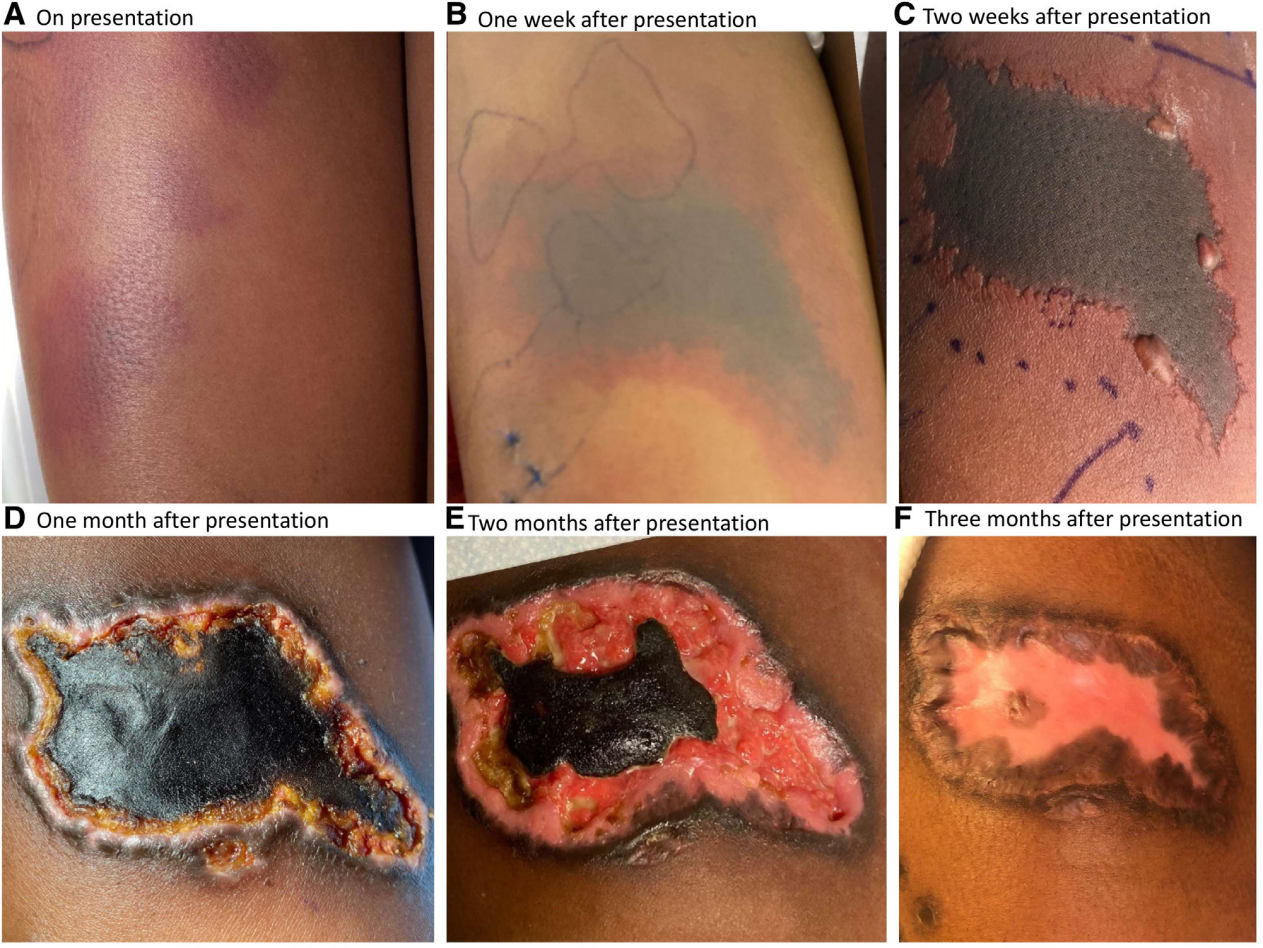
Anterior views of the right thigh on presentation (A), 1 week later (B), 2 weeks later (C), 1 month later (D), 2 months later (E), and 3 months later (F). Purpura (A,B) were not palpable as are observed in cutaneous small-vessel vasculitis. Note the absence of marked purulence (D, E) or cribriform scarring (F) which are observed in pyoderma gangrenosum.

**FIGURE 2.**
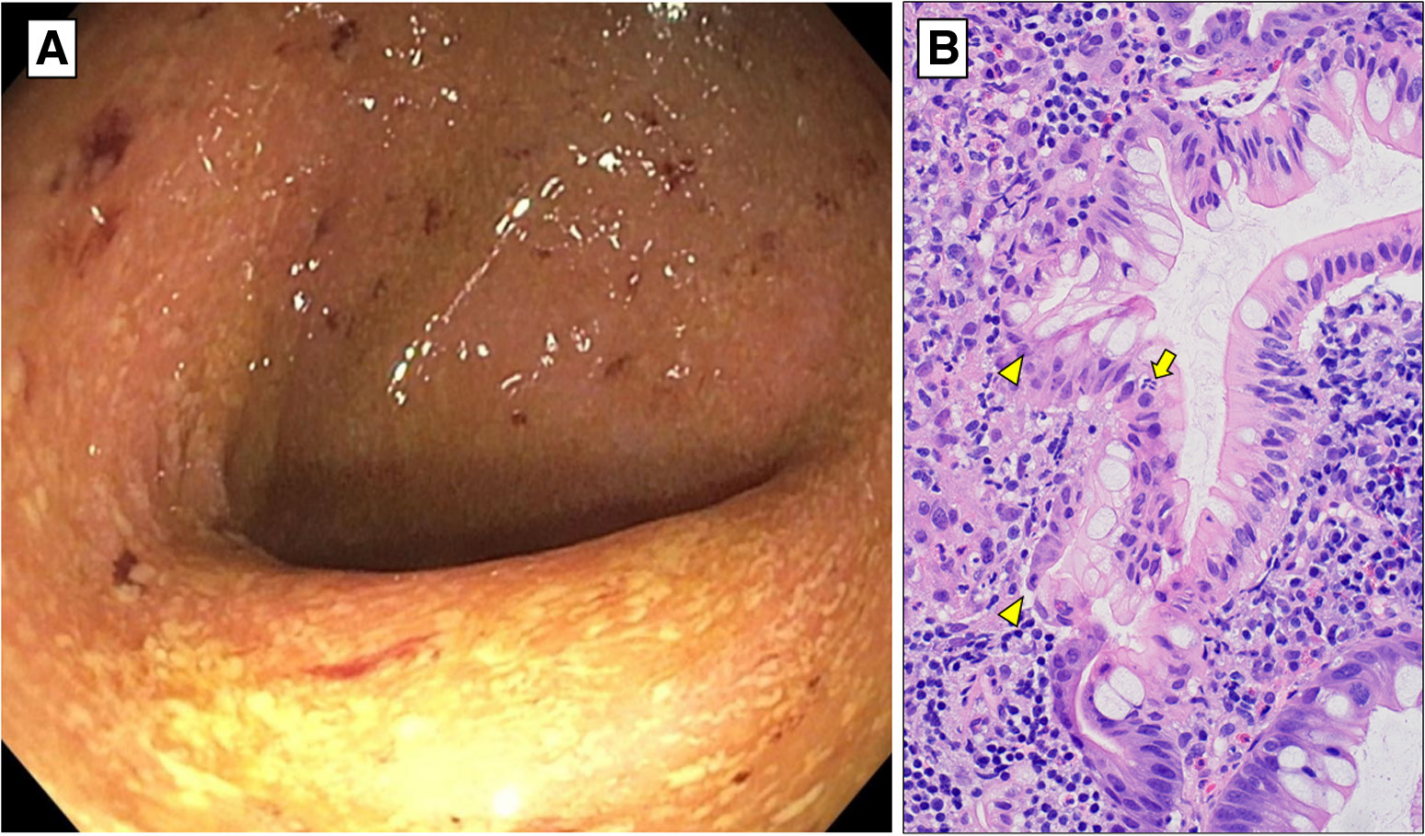
Colonoscopy showed gross pancolitis (A) with erythema, edema, friability and microhemorrhages. Histopathologic findings (B) included chronic active colitis with cryptitis (arrow) and abnormal crypt branching (arrowheads) but no granulomata.

A 7-year-old girl with sickle cell trait was admitted with inability to bear weight and a painful rash. Examination demonstrated retiform purpura (RP) of the right leg (Fig. 1A) with tenderness. The lesions had worsened despite treatment with cephalexin. Family history was unremarkable. There was no other skin involvement, history of fevers, or abnormalities on exam. Bloodwork was notable for microcytic anemia, elevated ESR, mildly elevated transaminases, and normal INR, albumin, and creatine kinase. MRI showed no muscle, bone, or joint involvement. Sequential cutaneous punch biopsies showed superficial and deep mixed perivascular and interstitial dermatitis without thromboses or leukocytoclastic vasculitis. Direct immunofluorescence findings were nonspecific. Hematologic evaluation included normal protein C and S levels, and mildly elevated d-dimer. Rheumatologic and infectious disease evaluations were also unremarkable. Over the following weeks new purpuric patches appeared, with some developing central duskiness and necrosis (Fig. 1B,C). After being noted to have mild colitic symptoms (PUCAI 25), elevated fecal calprotectin, persistently elevated transaminases, and a positive antinuclear antibody she underwent endoscopy, colonoscopy, and liver biopsy leading to the diagnoses of ulcerative colitis (Fig. 2) and autoimmune hepatitis. Treatment with systemic steroids improved her pain and transaminases, but the rash continued to evolve over the following 3 months developing a central necrotic eschar with surrounding ulceration to the subcutaneous tissue that slowly healed with supportive wound care (Fig. 1C–E).

RP presents as branching purpuric patches and develops when dermal and subcutaneous blood vessels are compromised by vessel wall damage or lumen occlusion causing pain, necrosis, and ulceration (1). Underlying processes include those that damage the vessel wall such as small- or medium-vessel vasculitides, angioinvasive infections, and depositional diseases. RP also results from vessel lumen occlusion due to thrombosis from hypercoagulable states, platelet disorders, and red blood cell occlusion, as well as embolic processes (1,2). Clinically, it can resemble cutaneous small-vessel vasculitis (CSVV) or pyoderma gangrenosum but is differentiated by the lack of palpable purpura or marked purulence, respectively (3,4). Histologically, the vascular origin of RP is deeper than CSVV and it lacks the neutrophilic infiltrate characteristic of pyoderma gangrenosum (3–5). Treatment should address the underlying etiology and include supportive wound care (6). Here, the vaso-occlusive process RP was the presenting feature in a patient with unrecognized inflammatory bowel disease. This may reflect the incompletely understood association between coagulation and inflammation in inflammatory bowel disease that is posited to result from the interplay between a hyperactive coagulation system, decreases in natural coagulation inhibitors, abnormal fibrinolysis, endothelial dysfunction, chronically activated platelets, and systemic inflammation (2,7,8).
